# Cultural adaptation of the Brazilian version of the New Scale of Sexual Satisfaction (NSSS-BR) for sexuality and HIV

**DOI:** 10.1590/0034-7167-2025-0287

**Published:** 2026-10-19

**Authors:** Henrique Ciabotti Elias, Ana Carolina Franco Corsino, Laelson Rochelle Milanês Sousa, Marcela Antonini, Sagrario Gomez-Cantarino, Elucir Gir, Renata Karina Reis

**Affiliations:** I Universidade de São Paulo, Ribeirão Preto School of Nursing. Ribeirão Preto, São Paulo, Brazil; II Universidade Estadual do Maranhão, Nursing Program. Coroatá, Maranhão, Brazil; III University of Castilla-La Mancha, Faculty of Physical Therapy, Nursing, and Occupational Therapy. Toledo, Spain

**Keywords:** Methodological Study, Consensus, HIV, Personal Satisfaction, Sexuality., Estudio Metodológico, Consenso, VIH, Satisfacción Personal, Sexualidad.

## Abstract

**Objective:**

To translate and cross-culturally adapt the New Scale of Sexual Satisfaction (NSSS-BR) into Brazilian Portuguese for people living with HIV (PLHIV).

**Methods:**

This was a five-stage methodological study: translation, expert review, back-translation, semantic analysis, and pre-testing. The sample included 10 raters, 43 PLHIV in the test, and 40 in the pretest, conducted between November and December 2023 at a nursing school and a specialized HIV/AIDS service in São Paulo. Inclusion criteria varied by stage and included clinical experience, scientific publications, language proficiency, legal age, and ongoing clinical care. Data were collected via REDCap® and analyzed using SPSS®.

**Results:**

The Content Validity Index (CVI) was satisfactory: Individual CVI ranging from 0.87 to 0.99, and Global CVI of 0.90. Most participants considered the scale understandable and appropriate.

**Conclusion:**

The Brazilian version has been adapted, is understandable, and is culturally equivalent to the original.

## RESUMEN

**Objetivo:**

traducir y adaptar transculturalmente al portugués brasileño la Nueva Escala de Satisfacción Sexual (NSSS-BR) para personas que viven con el VIH (PVVIH).

**Métodos:**

Se trató de un estudio metodológico en cinco etapas: traducción, revisión por expertos, retraducción, análisis semántico y prueba piloto. La muestra contó con 10 revisores, 43 PVHIV en la prueba y 40 en la prueba preliminar, realizadas entre noviembre y diciembre de 2023 en una escuela de enfermería y un servicio especializado en VIH/SIDA de São Paulo. Los criterios de inclusión variaron según la etapa e incluyeron experiencia clínica, producción científica, dominio de idiomas, mayoría de edad y seguimiento clínico. Los datos se recopilaron a través de REDCap® y se analizaron con SPSS®.

**Resultados:**

El Índice de Validez de Contenido (IVC) fue satisfactorio: IVC-Individual oscilando entre 0,87 y 0,99, e IVC-Global de 0,90. La mayoría de los participantes consideró que la escala era comprensible y adecuada.

**Conclusión:**

La versión brasileña ha sido adaptada, es comprensible y culturalmente equivalente a la original.

## INTRODUCTION

The sexual health of people living with HIV is still neglected, hindered by stigma, the need to disclose HIV status^(1)^, and the management of transmission risk^(2)^, all of which compromise sexual satisfaction^(3)^. Despite advances in treatment and prevention, care remains insufficient, requiring interventions that take into account sexual partnerships and the central role of health professionals in comprehensive care ^(4-6)^.

The WHO’s concept of sexual health (2006) relates to sexuality and was expanded by the Pan American Health Organization (PAHO) to include sexual satisfaction and the promotion of sexual rights. This positive approach aims to ensure sexual health and the right to express sexuality ^(7,8)^, serving as a potential predictor of quality of life for people living with HIV ^(10)^.

The assessment of sexuality in people living with HIV should take into account both clinical and non-clinical factors. Studies indicate that low sexual desire is the main difficulty among men ^(3)^, while among women ^(11)^, the prevalence of sexual dysfunction reaches 71.4%, with particular emphasis on disorders related to desire, arousal, orgasm, pain, lubrication, and sexual dissatisfaction.

Assessing the sexual satisfaction of people living with HIV is essential for strengthening sexual health. Considering biopsychosocial aspects enables comprehensive care, with evidence-based, multidisciplinary care ^(12)^ that promotes treatment adherence. The promotion of sexual health is encouraged by organizations such as the Association of HIV Medicine ^(13)^.

The use of scales to assess sexual satisfaction has been increasing in the literature ^(14-16)^. In sub-Saharan Africa, a scale was developed and validated among HIV-serodiscordant couples, showing that greater sexual frequency and relationship quality are associated with higher sexual satisfaction. Given its good psychometric properties, the authors recommend the validation of new scales to expand studies on sexual and reproductive health among people living with HIV ^(17)^.

The use of scales in nursing care can be an effective strategy for providing comprehensive care to people living with HIV ^(18)^. Another important point is the multifaceted stigma surrounding the approach to specific groups^(19)^. When addressing sexuality, it is essential to establish a bond of trust between the sender and receiver of information. A systematic review of stigma scales revealed that few utilized pre-testing and semantic analysis, despite the recommendation of these steps in the validation process for different populations ^(20)^.

Given the above, the question arose: What is the cultural validity of the New Scale of Sexual Satisfaction among Brazilians living with HIV?

## OBJECTIVE

To describe the process of semantic and item equivalence, the first stage of validating the New Scale of Sexual Satisfaction (NSSS) for people living with HIV into Brazilian Portuguese.

## METHODS

### Ethical considerations

The project was approved by the Research Ethics Committee of the Ribeirão Preto School of Nursing and began following this approval and authorization from the author of the scale for cultural validation. All participants in stages 2, 4, and 5 signed the Informed Consent Form (ICF), in accordance with Resolution 466/2012.

### Study design, period, and location

This is a methodological study for the cultural adaptation of the New Scale of Sexual Satisfaction (NSSS) proposed by Stulhofer, Busko, and Brouillard ^(14)^ for people living with HIV, conducted at two institutions in São Paulo (educational and healthcare) in 2023 and 2024, in five stages: translation (stage 1), evaluation by an expert committee (stage 2), back-translation (stage 3), semantic analysis (stage 4), and pre-testing (stage 5), as shown in Figure 1:

**Figure 1:**
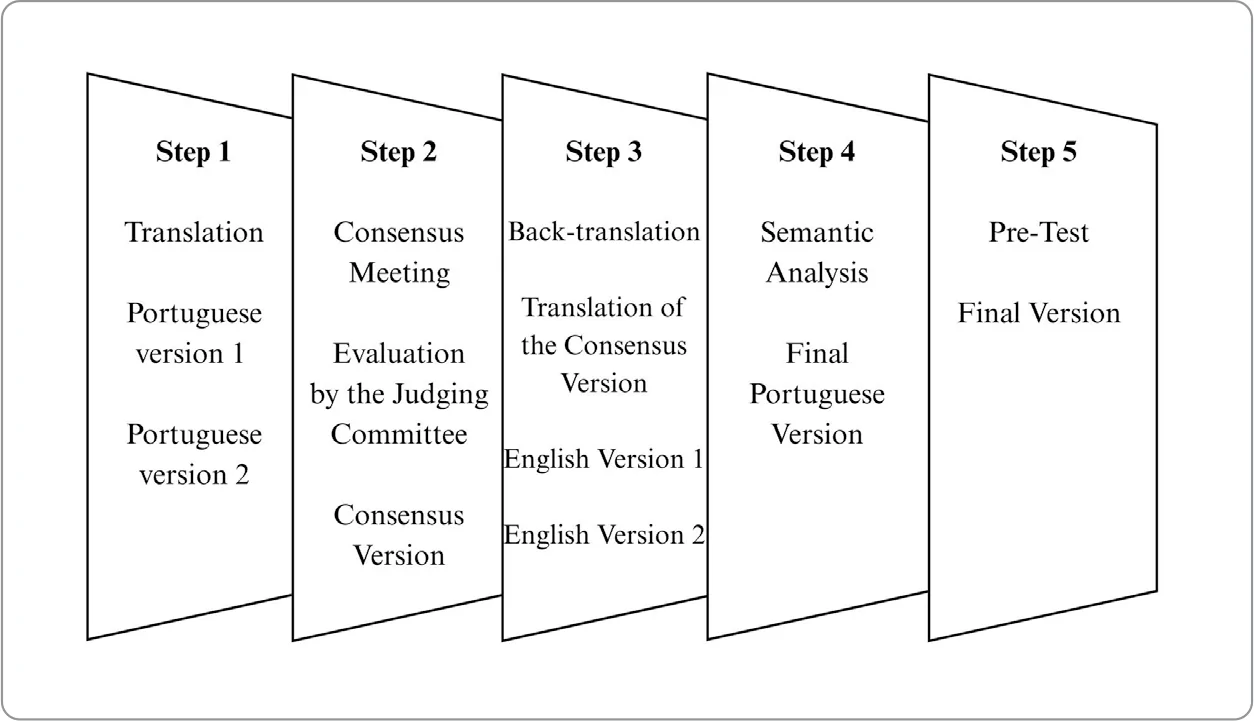
Methodological process used for the cultural adaptation of the NSSS. Ribeirão Preto, São Paulo, Brazil

To this end, the guidelines provided by Guillemin, Bombardier, and Beaton ^(21)^, Beaton et al. ^(22)^, and Polit & Beck ^(23)^ were used. For the statistical analyses, the guidelines provided by Polit & Beck^(23)^ were used.

### The New Scale for Sexual Satisfaction (NSSS)

The NSSS is a multidimensional scale consisting of 20 items designed to assess sexual satisfaction. Developed in 2010 in Croatia, the survey was validated in seven independent groups, including communities, clinics, and university students, with over 2,000 participants aged 18 to 55 in Croatia and the United States. The scale in question consists of two factors: individual, interpersonal, and behavioral perspectives. The first factor covers items 1 through 10 and relates to the individual lens. The second factor, in turn, focuses on the interpersonal and behavioral lens, assessed by items 11 through 20. This assessment is based on a five-dimensional conceptual model, which includes: sexual sensations, sexual awareness and focus, sexual exchange, emotional closeness, and sexual activity. The response scale used is a five-point Likert scale, and the total score range of the scale varies from 20 to 100 points. The final NSSS score is calculated by summing the ratings given to the 20 items, allowing for the calculation of separate scores for each of its two subscales. As demonstrated in the literature, the higher the score, the greater the sexual satisfaction. Internal reliability was 0.94, indicating excellent internal consistency of the total scale; test-retest reliability (approximately 1 month) was considered satisfactory in both samples (Croatia and the US), although it was not although this was not demonstrated in the original study ^(14)^.

### Procedures for Cross-Cultural Adaptation

#### Step 1 - Translation of the NSSS

In this step, the NSSS was translated from English into Brazilian Portuguese by two independent, certified translators with no prior knowledge of the study, following the recommendations of Guillemin Bombardier and Beaton^(21)^. After contacting the translators and agreeing on the budget and deadline, two translated versions were obtained, named Portuguese Version 1 and Portuguese Version 2, with data collection taking place in April 2023. A comparative analysis of these translations was conducted in the next stage.

#### Step 2 - Evaluation by the expert committee

In this step, the certified translations of the NSSS were evaluated by a panel of judges for clarity, appropriateness, and relevance, following the recommendations of Polit & Beck^(23)^ and Marques & Freitas^(24)^. The committee was formed based on the following inclusion criteria: clinical experience with people living with HIV, published research in the field, or membership in the target population (people living with HIV), as well as knowledge of linguistics (Portuguese and English); and exclusion criteria: judges and translators who did not complete the translation and back-translation stages^(22)^. The meeting took place at a nursing school in São Paulo to analyze the translated versions and the original, discussing each item individually to select the best translation. Each expert also individually evaluated the items via questionnaires on the REDCap® platform, using a Likert scale and space for comments. The meeting was held at a São Paulo higher education institution and lasted approximately 90 minutes, resulting in a consensus version of the scale in Portuguese, collected in June 2023. For data analysis, adapted Delphi ^(25)^ and Nominal Group^(26)^ methods were applied, in addition to calculating the Content Validity Index (CVI) for each item and overall, considering a CVI ≥ 0.80 to be valid, as recommended by Polit & Beck^(23)^.

#### Step 3 - Back-translation

Following the consensus meeting, the consensus Portuguese version of the NSSS was back-translated into English by two certified translators-who were independent and different from those involved in the first step-without their knowledge of the research objectives or the original version. After negotiation, two back-translated versions were obtained, designated English Version 1 and 2, collected in September 2023. The original author of the scale reviewed these versions and approved the Final English Version.

#### Step 4 - Semantic Analysis of the Items

The objective of this stage was to assess the comprehensibility of the scale items, as recommended by Polit & Bec^(23)^, among people living with HIV, in accordance with the recommendations of Guillemin et al.^(21)^ and Beaton et al.^(22)^. The included participants were: individuals over 18 years of age and under clinical care; the exclusion criteria were: those who did not attend the appointments previously scheduled by the team, those who did not complete all questions on all evaluated questionnaires, incarcerated patients, and illiterate individuals. Participants were contacted based on the daily appointment schedules provided by the service. They were invited to participate after their medical appointments in a private room within the service itself and answered semi-structured questionnaires adapted from other studies^(27,28)^ regarding sociodemographic characteristics and semantic analysis of the scale, collected between November and December 2023 via the REDCap® platform. The data were extracted from REDCap® and transferred to the Statistical Package for the Social Sciences (SPSS - version 22.0), where statistical analyses were performed: descriptive analyses (absolute and relative) of all questionnaires administered at this stage. No semantic changes were suggested by the target population at this stage.

#### Stage 5 - Pre-Test

In this stage, the recommendations of Guillermin, Bombardier, and Beaton^(21)^ were followed. After adaptation, the final version of the NSSS was administered to evaluate data collection performance at the same healthcare facility as in the previous stage. Participants included people living with HIV who were over 18 years of age and under clinical care, following inclusion and exclusion criteria similar to those of the previous stage. Data collection took place between December 2023 and January 2024, conducted by a researcher and a nursing student, with interviews lasting approximately 20 minutes. The data, collected via REDCap®, were analyzed in SPSS® using descriptive statistics.

## RESULTS

### Step 2: Consensus Meeting

Twelve curricula were reviewed, with 10 reviewers participating (100%); 5 (50%) participated online and 5 (50%) in person. The group was equally divided between men and women, ranging in age from 29 to 65 years (mean 42.6). Seven (70%) were healthcare professionals, including 3 (30%) faculty members, with 5 to 21 years of experience in HIV; 7 (70%) had publications in the field, and 3 (30%) had more than 15 years of experience as researchers. The group also included 2 nurses (20%), 2 sexologists (20%), 1 linguistics specialist (10%), 1 translator (10%), and 1 cultural producer representing people living with HIV (10%). Regarding clinical experience, 5 (50%) had it, 4 (40%) did not, and 1 (10%) did not respond (Table 1).

**Table 1:** Content validity index by item and overall for the version reviewed by experts (n=10) and the Brazilian version of the New Scale for Sexual Satisfaction for people living with HIV. Ribeirão Preto, São Paulo, Brazil, 2025

Items	Items Translated from the New Scale of Sexual Satisfaction for People Living with HIV	IVC - I^*^			Consensus version of the New Scale of Sexual Satisfaction for people living with HIV
		Attributes			
		Clarity	Relevance	Relevance	
	Pense na sua vida sexual durante os últimos seis meses, por favor, e classifique sua satisfação sexual nos últimos seis meses, nos seguintes aspectos:	0.9	0.9	0.9	Pense na sua vida sexual durante os últimos seis meses, por favor, e classifique sua satisfação sexual nos últimos seis meses, nos seguintes aspectos
**1**	A intensidade da minha excitação sexual.	1	1	1	A intensidade da minha excitação sexual.
**2**	A qualidade dos meus orgasmos.	1	1	1	A qualidade dos meus orgasmos.
**3**	“Me soltar” e me entregar ao prazer durante o sexo.	0.9	1	1	“Me soltar” e me entregar ao prazer durante o sexo.
**4**	Meu foco/concentração durante a atividade sexual.	0.9	1	1	Meu foco/concentração durante a atividade sexual.
**5**	A maneira como eu reajo sexualmente a minha parceria.	0.9	1	1	A maneira como eu reajo sexualmente a minha parceria.
**6**	O funcionamento sexual do meu corpo.	0.8	1	0.9	O funcionamento sexual do meu corpo.
**7**	Minha abertura emocional na relação sexual.	0.9	1	1	Minha abertura emocional na relação sexual.
**8**	Meu humor após a atividade sexual.	0.9	1	1	Meu humor após a atividade sexual.
**9**	A frequência dos meus orgasmos.	0.9	1	1	A frequência dos meus orgasmos.
**10**	O prazer que eu proporciono a minha parceria.	0.8	1	1	O prazer que eu proporciono a minha parceria.
**11**	O meu equilíbrio entre dar e receber na relação sexual.	0.8	1	1	O meu equilíbrio entre dar e receber na relação sexual.
**12**	A abertura emocional da minha parceria durante o sexo.	0.9	1	1	A abertura emocional da minha parceria durante o sexo.
**13**	O início da atividade sexual por parte da minha parceria.	0.9	0.8	1	O início da atividade sexual por parte da minha parceria.
**14**	A capacidade da minha parceria de ter orgasmos.	0.9	1	1	A capacidade da minha parceria de ter orgasmos.
**15**	A entrega da minha parceria ao prazer sexual (“se soltar”).	0.8	1	1	A entrega da minha parceria ao prazer sexual (“se soltar”).
**16**	A maneira como minha parceria cuida de minhas necessidades sexuais.	0.9	1	0.9	A maneira como minha parceria cuida de minhas necessidades sexuais.
**17**	A criatividade sexual da minha parceria.	0.9	1	1	A criatividade sexual da minha parceria.
**18**	A disponibilidade sexual da minha parceria.	0.9	1	1	A disponibilidade sexual da minha parceria.
**19**	A variedade das minhas atividades sexuais.	0.9	0.9	1	A disponibilidade sexual da minha parceria.
**20**	A frequência da minha atividade sexual.	1	1	1	A frequência da minha atividade sexual.
	NSSS Response Categories	1	1	1	
	IVC-G^**^	0.87	0.99	0.99	

* *Content Validity Index for each item;*

* ^*^Overall Content Validity Index

Among the specialists, 8 (80%) do not have a specialization in HIV, while 2 (20%) do. Regarding academic qualifications, 3 (30%) hold a Ph.D., 2 (20%) are associate professors, 2 (20%) hold a master’s degree, 2 (20%) have a specialization, and 1 (10%) has completed higher education. In HIV research, 7 (70%) work in the field, and 5 (50%) have defended a thesis or dissertation on the topic. The meeting lasted approximately 1 hour and 30 minutes. Following the Delphi and adapted Nominal Group methods, items 1, 2, 4, 8, 9, 11, 12, 14, 17, 18, 20, and the response categories received no suggestions from the judges. The content validity indices (CVI) were high: CVI-I of 0.87 for clarity, 0.98 for relevance, and 0.99 for importance, with a total CVI-G of 1.00, indicating excellent agreement.

### Step 4: Semantic analysis of the items

Table 2 presents the results of the sociodemographic profile of the 43 participants in the NSSS pre-test phase.

**Table 2:** Profile of the 43 participants in the Semantic Analysis, Ribeirão Preto, São Paulo, Brazil, 2025

Variable	n (%)
**Age group (full years)**	
20 a 60	38(88.5)
Over 60	05 (11.5)
**Skin color**	
White	20 (46.5)
Not white	23 (53.5)
**Religion**	
Catholic	18 (41.9)
Not catholic	28 (58.1)
**Gender identity**	
Cisgender man	32 (74.4)
Cisgender women	10 (23.3)
Transgender Woman	01 (2.3)
**Sexual Orientation**	
Gay	22 (51.2)
Heterosexual	18 (41.9)
Bisexual	03 (7.0)
**Marital Status**	
Single / Divorced	27 (62.8)
Married	14 (32.5)
Widows and widowers and others	02 (4.6)
**Education**	
Elementary Education	14 (32.6)
Elementary School	05 (11.7)
High School	14 (32.6)
Undergraduate Program	07 (16.3)
Graduate studies/residency/specialization	03 (7.0)
**Employment status**	
Full-time job	01 (2.3)
Self-employed	14 (30.1)
Ongoing informal work (e.g., street vendor, off-the-books work, etc.)	09 (21.0)
Retired, Recipient of benefits/pensioner	15 (34.9)
Unemployed	02 (4.7)
Sex worker	03 (7.0)
Income^*^	
≤ 2	23 (53.3)
≥ 2	20 (46.4)

* *Minimum wage (MW) in Brazil at the time of data collection (2023-2024): R$1,412.00 (Brazil, 2023)*

*link: https://www.gov.br/planalto/pt-br/acompanhe-o-planalto/noticias/2023/12/salariominimo-de-2024-tera-ganho-real-e-crescera-3pp-alem-dos-3-85-da-inflacao*

Of the 43 participants, 40 (93.0%) rated the overall scale as good, and 3 (7.0%) as fair. Regarding their understanding of the questions, 30 (69.8%) found them easy, 11 (25.6%) found them fair, and 2 (4.7%) found them difficult. The Likert scale caused difficulty for 9 (20.9%) people, while 30 (69.8%) had no problems. In addition, 42 (97.7%) considered the questions important for sexual satisfaction.

In the open-ended question about possible changes, 20 (46.6%) answered “no” and 19 (44.2%) “nothing to report,” indicating a lack of semantic suggestions. Upon analyzing the items individually, comprehension difficulties were noted in items 1, 2, 5, 9, 10, 11, 12, 13, 14, 18, 19, and 20. However, at least 40 participants reported no difficulty with any item; 26 (60.4%) did not request rephrasing, and 17 (39.6%) made no additional comments. No changes were suggested in the semantic analysis.

### Step 5 - Pre-test

Table 3 presents the results of the sociodemographic profile of the 40 individuals who participated in the pre-test phase of the NSSS.

**Table 3:** Profile of the 40 individuals who participated in the pre-test, Ribeirão Preto, São Paulo, Brazil, 2025

Variable	n (%)
**Age group (full years)**	
20 a 60	38 (95.0)
≥ 60	02 (5.0)
**Skin color**	
White	27 (67.5)
Not white	13 (32.5)
**Religion**	
Catholic	10 (25.0)
Not catholic	40 (75.0)
**Gender identity**	
Cisgender man	29 (72.5)
Cisgender women	09 (22.5)
Transgender Woman	02 (5.0)
**Sexual Orientation**	
Gay	23 (57.5)
Heterosexual	12 (30.0)
Bisexual	03 (7.5)
Another	02 (5.0)
**Marital Status**	
Single / Divorced	26 (65.0)
Married	12 (30.0)
Widows and widowers and others	02 (5.0)
**Education**	
Elementary School	23 (57.5)
High School	13 (32.5)
Undergraduate	03 (7.5)
**Employment status**	
Employed	14 (35.0)
Self-employed	15 (37.5)
Retired, receiving benefits/pension	06 (15.0)
Unemployed	04 (10.0)
Student (on scholarship)	01 (2.5)
Income^*^	
≤ 2	22 (55.0)
≥ 2	18 (45.0)

* *Minimum wage (MW) in Brazil at the time of data collection (2023-2024): R$1,412.00 (Brazil, 2023)*

*link: https://www.gov.br/planalto/pt-br/acompanhe-o-planalto/noticias/2023/12/salariominimo-de-2024-tera-ganho-real-e-crescera-3pp-alem-dos-3-85-da-inflacao*

Most responses on the NSSS ranged from “moderately satisfied” to “extremely satisfied.” Item 17, “My partner’s sexual creativity,” had the highest percentage of “not at all satisfied” responses (4 participants; 10%). Item 13, “My partner’s initiative in initiating sexual activity,” had the highest frequency of “somewhat satisfied” responses (7 participants; 17.5%). The distribution of responses from people living with HIV regarding the NSSS items is presented in Table 4.

**Table 4:** Distribution of responses from people living with HIV (n=40) collected during the pre-test phase of the New Scale of Sexual Satisfaction, Ribeirão Preto, São Paulo, Brazil, 2025

Scale item	Not at all satisfied	Not very satisfied	Moderately Satisfied	Very Satisfied	Extremely Satisfied
	n (%)	n (%)	n (%)	n (%)	n (%)
**1.** *A intensidade da minha excitação sexual.*	03 (7.5)	00 (40.0)	16 (35.0)	14 (35.0)	07 (17.5)
**2.** *A qualidade dos meus orgasmos.*	02 (5.0)	01 (2.5)	12 (30.0)	21 (52.5)	04 (10.0)
**3.** *“Me soltar” e me entregar ao prazer durante o sexo.*	03 (7.5)	04 (10.0)	06 (15.0)	12 (30.00)	15 (37.5)
**4.** *Meu foco/concentração durante a atividade sexual.*	01 (2.5)	04 (10.0)	11 (27.5)	17 (42.5)	07 (17.5)
**5.** *A maneira como reajo sexualmente a minha parceria.*	02 (5.0)	00 (0.0)	09 (22.5)	20 (50.0)	09 (22.5)
**6.** *O funcionamento sexual do meu corpo.*	01 (2.5)	02 (5.0)	13 (32.5)	14 (35.0)	10 (25.0)
**7.** *Minha abertura emocional na relação sexual.*	02 (5.0)	02 (5.0)	13 (32.5)	16 (40.0)	07 (17.5)
**8.** *Meu humor após a atividade sexual.*	00 (0.0)	04 (10.0)	07 (17.5)	15 (37.5)	14 (35.0)
**9.** *A frequência dos meus orgasmos.*	03 (7.5)	01 (2.5)	12 (30.0)	17 (42.5)	07 (17.5)
**10.** *O prazer que proporciono a minha parceria.*	01 (2.5)	02 (5.0)	13 (32.5)	13 (32.5)	11 (27.5)
**11.** *O meu equilíbrio entre dar e receber na relação sexual.*	01 (2.5)	01 (2.5)	15 (37.5)	17 (42.5)	06 (15.0)
**12.** *A abertura emocional da minha parceria durante o sexo.*	01 (2.5)	01 (2.5)	15 (37.5)	13 (32.5)	10 (25.0)
**13.** *O início da atividade sexual por parte da minha parceria.*	02 (5.0)	07 (17.5)	09 (22.5)	14 (35.0)	08 (20.0)
**14.** *A capacidade da minha parceria de ter orgasmos.*	01 (2.5)	03 (7.5)	08 (20.0)	14 (35.0)	14 (35.0)
**15.** *A entrega da minha parceria ao prazer sexual (“se soltar”).*	02 (5.0)	02 (5.0)	10 (25.0)	13 (32.5)	13 (32.5)
**16.** *A maneira como minha parceria cuida de minhas necessidades sexuais.*	02 (5.0)	05 (12.5)	10 (25.0)	09 (22.5)	14 (35.0)
**17.** *A criatividade sexual da minha parceria.*	04 (10.0)	03 (7.5)	12 (30.0)	12 (30.0)	09 (22.5)
**18.** *A disponibilidade sexual da minha parceria.*	03 (7.5)	04 (10.0)	10 (25.0)	12 (30.0)	11 (27.5)
**19.** *A variedade das minhas atividades sexuais.*	03 (7.5)	07 (17.5)	08 (20.0)	14 (35.0)	08 (20.0)
**20.** *A frequência da minha atividade sexual.*	05 (12.5)	05 (12.5)	12 (30.0)	06 (15.0)	12 (30.0)

## DISCUSSION

This study translated and adapted the New Sexual Satisfaction Scale for people living with HIV, creating a reliable tool that assesses sexual satisfaction in this population, thereby helping healthcare professionals provide more humane and effective care.

In the validation of scales, the participation of experts with relevant training and experience is essential, especially in studies involving people living with HIV. Rigorous content evaluation regarding relevance and appropriateness ensures the quality of the instrument. Studies such as those by Kim et al.^(29)^ and Yafi et al.^(30)^ reinforce the importance of critical analysis and standardization for the scale’s future clinical use.

When translating previously developed scales, cultural discrepancies may arise regarding specific terms, making the participation of experts in the target language and translators in the analysis essential. During the translation of the NSSS into Brazilian Portuguese, terminological adjustments were necessary, as was the case in the adaptation into Chinese ^(16)^, to ensure comprehension by the target population.

Translation and back-translation by independent translators are essential steps in the validation of scales. The NSSS underwent this process for Brazilian Portuguese, as did the Female Sexual Function Index for Breast Cancer Patients (FSFI-BC), validated for women with breast cancer, which demonstrated excellent content and internal reliability ^(31)^.

The profile of the participants in this study, particularly regarding educational attainment, is similar to that found in studies on stigma and HIV in Brazil. Educational attainment proved to be an important factor in understanding the scale items. In both stages, a significant proportion of participants had not completed high school, which reinforces the importance of adapting instruments to the educational reality of the target population ^(32)^.

In the semantic analysis, 51.2% of participants identified as homosexual, and in the test, 57.5%. People living with HIV who are gay or bisexual face double stigma, as observed in Lebanon, where 73.4% reported at least one mental disorder ^(33)^. The inclusion of sexual and gender diversity in the validation of scales is essential to ensure inclusive and effective instruments ^(34)^.

### Limitations of the study

The study employed appropriate methodologies but had limitations, such as convenience sampling conducted at a single facility, which precluded the analysis of invariance across groups. Validation across different cultural contexts requires the scale to be applied in various settings, with larger and more representative samples. Furthermore, because it was conducted exclusively with this population, the scale requires additional validation for other audiences. Although recognizing the need for future validations is important, the study was limited to analyzing semantic and content validity, failing to address fundamental stages of the psychometric process, such as construct validity, factor validity, validity related to other variables, internal consistency, and empirical testing. It is also important to consider the profile of the sample of experts involved and the limitations regarding the generalizability of the results, even among people living with HIV.

### Contributions to the fields of nursing, health, or public policy

The instrument provides a tool for assessing the sexual satisfaction of people living with HIV, which contributes to a more comprehensive and humanized approach to sexuality by these professionals.

## CONCLUSION

The New Scale of Sexual Satisfaction has demonstrated content validity, as confirmed by expert review, and semantic validity, as verified through pretesting with the target population. It is recommended that future studies investigate its clinical applicability in different cultural contexts in order to consolidate its evidence-based use.

## Data Availability

Not applicable.
