# Supplementary material for: Cultural adaptation of the Brazilian version of the New Scale of Sexual Satisfaction (NSSS-BR) for sexuality and HIV
Source: Rev Bras Enferm. 2026 Oct 19;79:e20250287. doi: 10.1590/0034-7167-2025-0287 (PMC13679584; doi:10.1590/0034-7167-2025-0287)
Supplement: Translation

# Translation: Adaptação cultural da versão brasileira da *New Scale Satisfaction Sexual* (NSSS-BR) para sexualidade e HIV

**DOI:** 10.1590/0034-7167-2025-0287pt

**Authors:** Henrique Ciabotti Elias, Ana Carolina Franco Corsino, Laelson Rochelle Milanês Sousa, Marcela Antonini, Sagrario Gomez-Cantarino, Elucir Gir, Renata Karina Reis

**Affiliations:** I Universidade de São Paulo, Escola de Enfermagem de Ribeirão Preto. Ribeirão Preto, São Paulo, Brasil; II Universidade Estadual do Maranhão, Curso de Enfermagem. Coroatá, Maranhão, Brasil; III Universidade de Castilla-La Mancha, Facultad de Fisioterapia, Enfermería y Terapia Ocupacional. Toledo, Espanha

**Keywords:** Estudo Metodológico, Consenso, HIV, Satisfação Pessoal, Sexualidade.

## Abstract

**Objetivo:**

traduzir e adaptar transculturalmente para o português brasileiro a *New Scale Satisfaction Sexual* (NSSS-BR) para pessoas vivendo com HIV (PVHIV).

**Métodos:**

Tratou-se de um estudo metodológico com cinco etapas: tradução, avaliação por especialistas, retrotradução, análise semântica e pré-teste. A amostra contou com 10 juízes, 43 PVHIV no teste e 40 no pré-teste, realizados entre novembro e dezembro de 2023 em uma escola de enfermagem e um serviço especializado em HIV/AIDS paulista. Os critérios de inclusão variaram conforme a etapa e incluíram experiência clínica, produção científica, domínio de idiomas, maioridade e acompanhamento clínico. Os dados foram coletados via REDCap® e analisados com o SPSS®.

**Resultados:**

O Índice de Validade de Conteúdo (IVC) foi satisfatório: IVC-Individual variando entre 0,87 e 0,99, e IVC-Global de 0,90. A maioria dos participantes considerou a escala compreensível e adequada.

**Conclusão:**

A versão brasileira foi adaptada, é compreensível e culturalmente equivalente à original.

## INTRODUÇÃO

A saúde sexual de pessoas vivendo com HIV ainda é negligenciada, sendo prejudicada por estigmas, necessidade de revelar a sorologia^(1)^ e gerenciamento do risco de transmissão^(2)^, o que compromete a satisfação sexual^(3)^. Apesar dos avanços em tratamento e prevenção, a assistência permanece insuficiente, exigindo intervenções que considerem as parcerias sexuais e o papel central dos profissionais de saúde no cuidado integral ^(4-6)^.

O conceito de saúde sexual da OMS (2006) relaciona-se à sexualidade e foi ampliado pela Organização Pan Americana de Saúde (OPAS), incluindo a satisfação sexual e a promoção dos direitos sexuais. Essa abordagem positiva visa garantir saúde sexual e o direito à expressão da sexualidade^(7,8)^, sendo um potencial preditor da qualidade de vida de pessoas vivendo com HIV^(10 )^.

A avaliação da sexualidade em pessoas vivendo com HIV deve considerar fatores clínicos e não clínicos. Estudos apontam o baixo desejo sexual como a principal dificuldade entre homens ^(3)^, enquanto, entre mulheres ^(11)^, a prevalência de disfunção sexual chega a 71,4%, com destaque para transtornos do desejo, excitação, orgasmo, dor, lubrificação e insatisfação sexual.

Avaliar a satisfação sexual de pessoas vivendo com HIV é fundamental para fortalecer a saúde sexual. Considerar os aspectos biopsicossociais permite um cuidado integral, com assistência multiprofissional ^(12)^ orientada por evidências científicas, favorecendo a adesão ao tratamento. A promoção da saúde sexual é incentivada por entidades como a Associação de Medicina do HIV ^(13)^.

O investimento em escalas para avaliação da satisfação sexual tem crescido na literatura ^(14-16)^. Na África Subsaariana, uma escala foi desenvolvida e validada com casais sorodiferentes para o HIV, evidenciando que maior frequência sexual e qualidade do relacionamento estão associadas à maior satisfação sexual. Com boas propriedades psicométricas, os autores recomendam a validação de novas escalas para ampliar os estudos sobre saúde sexual e reprodutiva em pessoas vivendo com HIV^(17)^.

O uso de escalas na assistência de Enfermagem pode ser uma estratégia eficiente no cuidado integral a pessoas que vivem com o HIV ^(18)^. Outro ponto importante é o estigma multifacetado que envolve a abordagem em grupos específicos^(19)^. Na abordagem da sexualidade, é fundamental estabelecer vínculo de confiança entre emissor e receptor da informação. Revisão sistemática sobre escalas de estigma revelou que poucas utilizaram pré-teste e análise semântica, apesar da recomendação dessas etapas no processo de validação para diferentes populações ^(20)^.

Diante do exposto, surgiu a pergunta: qual a validade cultural da *New Scale Satisfaction Sexual* em pessoas que vivem com HIV brasileiras?

## OBJETIVO

Descrever o processo de equivalência semântica e de itens, primeira etapa da validação da *New Scale Satisfaction Sexual (NSSS)* em pessoas que vivem com HIV para o português brasileiro.

## MÉTODOS

### Aspectos éticos

O projeto foi aprovado pelo Comitê de Ética em Pesquisa da Escola de Enfermagem de Ribeirão Preto e teve início após essa aprovação e autorização do autor da escala para validação cultural. Todos os participantes das etapas 2, 4 e 5 assinaram o Termo de Consentimento Livre e Esclarecido (TCLE), conforme a Resolução 466/2012.

### Desenho, período e local do estudo

Trata - se de estudo metodológico para adaptação cultural da *New Scale Satisfaction Sexual* (NSSS) proposta por Stulhofer, Busko, Brouillard^(14)^ para pessoas que vivem com HIV, realizado em duas instituições paulistas (educacional e assistencial), em 2023 e 2024, em cinco etapas: tradução (etapa 1), avaliação por comitê de peritos (etapa 2), retrotradução (etapa 3), análise semântica (etapa 4) e pré-teste (etapa 5), conforme a Figura 1:

**Figura 1:** Processo metodológico utilizado para adaptação cultural da NSSS. Ribeirão Preto, São Paulo, Brasil

Para tanto foi utilizado o referencial de Guillemin, Bombardier, Beaton^(21)^, Beaton et al. ^(22)^ e Polit & Beck ^(23)^. Para as análises estatísticas foi utilizado o referencial de Polit & Beck^(23)^.

### A Escala *New Scale Satisfaction Sexual (NSSS)*

A NSSS é uma escala multidimensional composta por 20 itens cujo propósito é avaliar a satisfação sexual. Desenvolvida em 2010 na Croácia, a pesquisa foi validada em sete grupos independentes, incluindo comunidades, clínicas e universitários, com mais de 2.000 participantes entre 18 e 55 anos, na Croácia e nos Estados Unidos. A escala em questão é composta por dois fatores: lente individual, interpessoal e behaviorista. O primeiro fator abrange os itens de número 1 a 10 e está relacionado a lente individual. O segundo fator, por sua vez, centra-se na lente interpessoal e behaviorista, sendo avaliado dos itens 11 a 20. Essa avaliação se fundamenta em um modelo conceitual de cinco dimensões, que incluem: sensações sexuais, consciência e foco sexual, troca sexual, proximidade emocional e atividade sexual. A escala de resposta adotada é do tipo Likert, com cinco pontos, e o intervalo de pontuação total da escala varia de 20 a 100 pontos. A pontuação final da NSSS é calculada mediante a soma das avaliações dadas aos 20 itens, possibilitando a obtenção de escores isolados para cada uma de suas duas subescalas. Conforme demonstrado na literatura, quanto maior a pontuação, maior a satisfação sexual. A confiabilidade interna foi de 0,94 indicando excelente consistência interna da escala total, a estabilidade temporal (teste-reteste de cerca de 1 mês) foi considerada satisfatória em ambas as amostras (Croácia e EUA), embora não tenha sido demonstrada no estudo original ^(14)^.

### Procedimentos para a Adaptação Transcultural

#### Etapa 1 - Tradução da NSSS

Nesta etapa, a NSSS foi traduzida do inglês para o português brasileiro por dois tradutores juramentados, independentes e sem conhecimento prévio da pesquisa, seguindo as recomendações de Guillemin Bombardier, Beaton^(21)^. Após contato e acordo sobre orçamento e prazo, foram obtidas duas versões traduzidas, denominadas Versão Português 1 e Versão Português 2, com a coleta realizada em abril de 2023. A análise comparativa dessas traduções ocorreu na etapa seguinte.

#### Etapa 2 - Avaliação pelo comitê de perito

Nesta etapa, as traduções juramentadas da NSSS foram avaliadas por um comitê de juízes quanto à clareza, pertinência e relevância, seguindo recomendações de Polit & Beck ^(23)^ e Marques & Freitas ^(24)^. O comitê foi formado com base nos critérios de inclusão: experiência clínica em pessoas que vivem com o HIV, pesquisas publicadas na área ou participante da população de interesse (pessoas que vivem com o HIV), e conhecimentos sobre linguística (português e inglês); e, exclusão: juízes e tradutores que não corresponderam as etapas de tradução e retrotradução ^(22)^. A reunião ocorreu em uma escola de enfermagem paulista para analisar as versões traduzidas e a original, discutindo item por item para escolher a melhor tradução. Cada perito também avaliou individualmente os itens via questionários na plataforma REDCap®, usando escala Likert e espaço para comentários. A reunião foi realizada em uma instituição educacional paulista de nível superior e durou cerca de 90 minutos e resultou em uma versão consensual da escala em português, coletada em junho de 2023. Para análise dos dados, foram aplicados métodos Delphi ^(25)^ e Grupo Nominal^(26)^ adaptados, além do cálculo do Índice de Validade de Conteúdo (IVC) para cada item e global, considerando válido IVC ≥ 0,80, conforme preconizado por Polit & Beck^(23)^.

#### Etapa 3 - Retrotradução (Back-translation)

Após a reunião de consenso, a versão consensual em português da NSSS foi retrotraduzida para o inglês por dois tradutores juramentados, independentes e distintos dos da primeira etapa, sem conhecimento dos objetivos da pesquisa ou da versão original. Após negociação, foram obtidas duas versões retrotraduzidas, denominadas Versão Inglês 1 e 2, coletadas em setembro de 2023. O autor original da escala analisou essas versões e aprovou a Versão Inglês Final.

#### Etapa 4 - Análise Semântica dos itens

O objetivo desta etapa foi verificar a compreensão dos itens da escala, conforme preconizado por Polit & Beck ^(23)^, por pessoas que vivem com HIV, conforme recomendações de Guillemin et al. ^(21)^ e Beaton et al. ^(22)^. Os participantes incluídos foram: maiores de 18 anos e em acompanhamento clínico, e os critérios de exclusão foram: aqueles que não compareceram as consultas agendadas previamente pela equipe, aqueles que não preencheram todas as perguntas de todos os questionários avaliados, pacientes que estavam privados de liberdade e analfabetos. Os participantes eram contatados com base nas agendas diárias de consulta oferecidas pelo serviço. Eram convidados a participar, após as consultas médicas, em sala privativa no próprio serviço, e responderam questionários semiestruturados adaptados de outros estudos^(27,28)^ sobre características sociodemográficas e análise semântica da escala, coletados entre novembro e dezembro de 2023 via plataforma REDCap®. Os dados foram extraídos do REDCap® e transportados para o *Statistical Package for the Social Sciences* (SPSS - versão 22.0) ® e realizadas as análises estatísticas - análises descritivas (absoluta e relativa) de todos os questionários aplicados nesta etapa. Não houve mudanças semânticas sugeridas pela população-alvo nesta etapa.

#### Etapa 5 - Pré Teste

Nesta etapa, as recomendações de Guillermin; Bombardier; Beaton ^(21)^ foram seguidas. Após a adaptação, a versão final da NSSS foi aplicada para avaliar o desempenho da coleta de dados no mesmo serviço de saúde da etapa anterior. Participaram pessoas vivendo com HIV e maiores de 18 anos e em acompanhamento clínico, seguindo critérios de inclusão e exclusão semelhantes ao da etapa anterior. A coleta ocorreu entre dezembro de 2023 e janeiro de 2024, conduzida por pesquisador e estudante de Enfermagem, com entrevistas de cerca de 20 minutos. Os dados, coletados via REDCap®, foram analisados no SPSS® por meio de estatísticas descritivas.

## RESULTADOS

### Etapa 2: Reunião de Consenso

Foram analisados 12 currículos, dos quais 10 juízes participaram (100%), sendo 5 (50%) online e 5 (50%) presencialmente. O grupo era igualmente dividido entre homens e mulheres, com idades entre 29 e 65 anos (média 42,6). Sete (70%) eram profissionais da saúde, incluindo 3 (30%) docentes, com 5 a 21 anos de experiência em HIV; 7 (70%) tinham publicações na área e 3 (30%) mais de 15 anos como pesquisadores. O grupo também incluía 2 enfermeiras (20%), 2 médicos sexólogos (20%), 1 especialista em linguística (10%), 1 tradutor (10%) e 1 produtor cultural representando pessoas que vivem com o HIV (10%). Quanto à experiência clínica, 5 (50%) tinham, 4 (40%) não, e 1 (10%) não respondeu (Tabela 1).

**Tabela 1:** Índice da Validade de Conteúdo por item e global da versão analisada pelos especialistas (n=10) e a versão brasileira da *New Scale Satisfaction Sexual* para pessoas que vivem com o HIV. Ribeirão Preto, São Paulo, Brasil, 2025

Itens	Itens Traduzidos da *New Scale* *Satisfaction Sexual* para PVHIV	IVC - I^*^			Versão consensual da *New Scale* *Satisfaction Sexual* para PVHIV
		Atributos			
		Clareza	Pertinência	Relevância	
	Pense na sua vida sexual durante os últimos seis meses, por favor, e classifique sua satisfação sexual nos últimos seis meses, nos seguintes aspectos:	0,9	0,9	0,9	Pense na sua vida sexual durante os últimos seis meses, por favor, e classifique sua satisfação sexual nos últimos seis meses, nos seguintes aspectos
**1**	A intensidade da minha excitação sexual.	1	1	1	A intensidade da minha excitação sexual.
**2**	A qualidade dos meus orgasmos.	1	1	1	A qualidade dos meus orgasmos.
**3**	“Me soltar” e me entregar ao prazer durante o sexo.	0,9	1	1	“Me soltar” e me entregar ao prazer durante o sexo.
**4**	Meu foco/concentração durante a atividade sexual.	0,9	1	1	Meu foco/concentração durante a atividade sexual.
**5**	A maneira como eu reajo sexualmente a minha parceria.	0,9	1	1	A maneira como eu reajo sexualmente a minha parceria.
**6**	O funcionamento sexual do meu corpo.	0,8	1	0,9	O funcionamento sexual do meu corpo.
**7**	Minha abertura emocional na relação sexual.	0,9	1	1	Minha abertura emocional na relação sexual.
**8**	Meu humor após a atividade sexual.	0,9	1	1	Meu humor após a atividade sexual.
**9**	A frequência dos meus orgasmos.	0,9	1	1	A frequência dos meus orgasmos.
**10**	O prazer que eu proporciono a minha parceria.	0,8	1	1	O prazer que eu proporciono a minha parceria.
**11**	O meu equilíbrio entre dar e receber na relação sexual.	0,8	1	1	O meu equilíbrio entre dar e receber na relação sexual.
**12**	A abertura emocional da minha parceria durante o sexo.	0,9	1	1	A abertura emocional da minha parceria durante o sexo.
**13**	O início da atividade sexual por parte da minha parceria.	0,9	0,8	1	O início da atividade sexual por parte da minha parceria.
**14**	A capacidade da minha parceria de ter orgasmos.	0,9	1	1	A capacidade da minha parceria de ter orgasmos.
**15**	A entrega da minha parceria ao prazer sexual (“se soltar”).	0,8	1	1	A entrega da minha parceria ao prazer sexual (“se soltar”).
**16**	A maneira como minha parceria cuida de minhas necessidades sexuais.	0,9	1	0,9	A maneira como minha parceria cuida de minhas necessidades sexuais.
**17**	A criatividade sexual da minha parceria.	0,9	1	1	A criatividade sexual da minha parceria.
**18**	A disponibilidade sexual da minha parceria.	0,9	1	1	A disponibilidade sexual da minha parceria.
**19**	A variedade das minhas atividades sexuais.	0,9	0,9	1	A disponibilidade sexual da minha parceria.
**20**	A frequência da minha atividade sexual.	1	1	1	A frequência da minha atividade sexual.
	Categoria de respostas da NSSS	1	1	1	
	IVC-G^**^	0,87	0,99	0,99	

^^*^^Índice de Validade de Conteúdo para cada Item;

* ^*^Índice de Validade de Conteúdo Global

Dos especialistas, 8 (80%) não têm especialização em HIV, enquanto 2 (20%) possuem. Quanto à formação acadêmica, 3 (30%) têm doutorado, 2 (20%) são livre-docentes, 2 (20%) possuem mestrado, 2 (20%) especialização e 1 (10%) ensino superior completo. Em pesquisa sobre HIV, 7 (70%) atuam na área, e 5 (50%) defenderam tese ou dissertação sobre o tema. A reunião durou cerca de 1h30min. Seguindo os métodos Delphi e Grupo Nominal adaptado, os itens 1, 2, 4, 8, 9, 11, 12, 14, 17, 18, 20 e as categorias de respostas não receberam sugestões dos juízes. Os índices de validade de conteúdo (IVC) foram altos: IVC-I de 0,87 para clareza, 0,98 para pertinência e 0,99 para relevância, com IVC-G total de 1,00, indicando excelente concordância.

### Etapa 4: Análise semântica dos itens

Na Tabela 2 estão demonstrados os resultados da caracterização sociodemográfica das 43 pessoas que participaram na fase do pré-teste da NSSS.

**Tabela 2:** Caracterização das 43 pessoas que participaram da Análise Semântica, Ribeirão Preto, São Paulo, Brasil, 2025

Variável	n (%)
**Faixa etária (anos completos)**	
20 a 60	38(88,5)
Maior que 60	05 (11,5)
**Cor de pele**	
Branca	20 (46,5)
Não branca	23 (53,5)
**Religião**	
Católico	18 (41,9)
Não católico	28 (58,1)
**Identidade de gênero**	
Homem Cisgênero	32 (74,4)
Mulher Cisgênero	10 (23,3)
Mulher Transgênero	01 (2,3)
**Orientação Sexual**	
Homossexual	22 (51,2)
Heterossexual	18 (41,9)
Bissexual	03 (7,0)
**Estado Civil**	
Solteiro (a) / Divorciado (a)	27 (62,8)
Casado (a)	14 (32,5)
Viúvo (a) e outros	02 (4,6)
**Escolaridade**	
Ensino Básico	14 (32,6)
Ensino Fundamental	05 (11,7)
Ensino Médio	14 (32,6)
Graduação	07 (16,3)
Pós graduação/ residência/ especialização	03 (7,0)
**Situação do trabalho**	
Carteira assinada	01 (2,3)
Autônomo	14 (30,1)
Trabalho informal contínuo (ex: camelô, contrato de gaveta, etc)	09 (21,0)
Aposentado, Benefício/ pensionista	15 (34,9)
Desempregado	02 (4,7)
Profissional do sexo	03 (7,0)
Renda ^^*^^	
≤ 2	23 (53,3)
≥ 2	20 (46,4)

* *Salário mínimo (SM) no Brasil na época da coleta (2023-2024): R$1.412, 00 (Brasil. 2023)*

*link: https://www.gov.br/planalto/pt-br/acompanhe-o-planalto/noticias/2023/12/salariominimo-de-2024-tera-ganho-real-e-crescera-3pp-alem-dos-3-85-da-inflacao*

Dos 43 participantes, 40 (93,0%) consideraram a escala geral como boa, e 3 (7,0%) como regular. Quanto à compreensão das questões, 30 (69,8%) as acharam fáceis, 11 (25,6%) regulares e 2 (4,7%) difíceis. A escala Likert causou dificuldade em 9 (20,9%) pessoas, enquanto 30 (69,8%) não tiveram problemas. Além disso, 42 (97,7%) consideraram as questões importantes para a satisfação sexual.

Na pergunta discursiva sobre possíveis mudanças, 20 (46,6%) responderam “não” e 19 (44,2%) “nada a declarar”, indicando ausência de sugestões semânticas. Ao analisar item por item, dificuldades de compreensão foram apontadas nos itens 1, 2, 5, 9, 10, 11, 12, 13, 14, 18, 19 e 20. Contudo, pelo menos 40 participantes não relataram dificuldade em nenhum item; 26 (60,4%) não desejavam reformulações e 17 (39,6%) não fizeram declarações adicionais. Não houve mudanças sugeridas na análise semântica.

### Etapa 5 - Pré teste

Na Tabela 3 estão demonstrados os resultados da caracterização sociodemográfica das 40 pessoas que participaram na fase do pré-teste da NSSS.

**Tabela 3:** Caracterização das 40 pessoas que participaram do pré-teste, Ribeirão Preto, São Paulo, Brasil, 2025

Variável	n (%)
**Faixa etária (anos completos)**	
20 a 60	38 (95,0)
≥ 60	02 (5,0)
**Cor de pele**	
Branca	27 (67,5)
Não branco	13 (32,5)
**Religião**	
Católico	10 (25,0)
Não católico	40 (75,0)
**Identidade de gênero**	
Homem Cisgênero	29 (72,5)
Mulher Cisgênero	09 (22,5)
Mulher Transgênero	02 (5,0)
**Orientação Sexual**	
Homossexual	23 (57,5)
Heterossexual	12 (30,0)
Bissexual	03 (7,5)
Outra	02 (5,0)
**Estado Civil**	
Solteiro (a) / Divorciado (a)	26 (65,0)
Casado (a)	12 (30,0)
Viúvo (a)	02 (5,0)
**Escolaridade**	
Ensino Fundamental	23 (57,5)
Ensino Médio	13 (32,5)
Graduação	03 (7,5)
**Situação do trabalho**	
Carteira assinada	14 (35,0)
Autônomo	15 (37,5)
Aposentado, Benefício/ pensionista	06 (15,0)
Desempregado	04 (10,0)
Estudante (bolsista)	01 (2,5)
Renda ^*^	
≤ 2	22 (55,0)
≥ 2	18 (45,0)

* *Salário mínimo (SM) no Brasil na época da coleta (2023-2024): R$1.412, 00 (Brasil. 2023)*

*link: https://www.gov.br/planalto/pt-br/acompanhe-o-planalto/noticias/2023/12/salariominimo-de-2024-tera-ganho-real-e-crescera-3pp-alem-dos-3-85-da-inflacao*

A maioria das respostas na NSSS variou entre “moderadamente satisfeito(a)” e “extremamente satisfeito(a)”. O item 17, “*A criatividade sexual da minha parceria*”, teve o maior percentual de respostas “nada satisfeito(a)” (4 participantes; 10%). Já o item 13, “*O início da atividade sexual por parte da minha parceria*”, apresentou a maior frequência de “pouco satisfeito” (7 participantes; 17,5%). A distribuição de respostas das pessoas que vivem com o HIV quanto aos itens da NSSS está apresentada na Tabela 4.

**Tabela 4:** Distribuição das respostas das pessoas que vivem com HIV (n=40) atribuídas na etapa do pré-teste da *New Scale Satisfaction Sexual,* Ribeirão Preto, São Paulo, Brasil, 2025

Item da escala	Nada satisfeito (a)	Pouco satisfeito (a)	Moderadamente Satisfeito (a)	Muito Satisfeito(a)	Extremamente Satisfeito (a)
	n (%)	n (%)	n (%)	n (%)	n (%)
**1.** A intensidade da minha excitação sexual.	03 (7,5)	00 (40,0)	16 (35,0)	14 (35,0)	07 (17,5)
**2.** A qualidade dos meus orgasmos.	02 (5,0)	01 (2,5)	12 (30,0)	21 (52,5)	04 (10,0)
**3.** “Me soltar” e me entregar ao prazer durante o sexo.	03 (7,5)	04 (10,0)	06 (15,0)	12 (30,00)	15 (37,5)
**4.** Meu foco/concentração durante a atividade sexual.	01 (2,5)	04 (10,0)	11 (27,5)	17 (42,5)	07 (17,5)
**5.** A maneira como reajo sexualmente a minha parceria.	02 (5,0)	00 (0,0)	09 (22,5)	20 (50,0)	09 (22,5)
**6.** O funcionamento sexual do meu corpo.	01 (2,5)	02 (5,0)	13 (32,5)	14 (35,0)	10 (25,0)
**7.** Minha abertura emocional na relação sexual.	02 (5,0)	02 (5,0)	13 (32,5)	16 (40,0)	07 (17,5)
**8.** Meu humor após a atividade sexual.	00 (0,0)	04 (10,0)	07 (17,5)	15 (37,5)	14 (35,0)
**9.** A frequência dos meus orgasmos.	03 (7,5)	01 (2,5)	12 (30,0)	17 (42,5)	07 (17,5)
**10.** O prazer que proporciono a minha parceria.	01 (2,5)	02 (5,0)	13 (32,5)	13 (32,5)	11 (27,5)
**11.** O meu equilíbrio entre dar e receber na relação sexual.	01 (2,5)	01 (2,5)	15 (37,5)	17 (42,5)	06 (15,0)
**12.** A abertura emocional da minha parceria durante o sexo.	01 (2,5)	01 (2,5)	15 (37,5)	13 (32,5)	10 (25,0)
**13.** O início da atividade sexual por parte da minha parceria.	02 (5,0)	07 (17,5)	09 (22,5)	14 (35,0)	08 (20,0)
**14.** A capacidade da minha parceria de ter orgasmos.	01 (2,5)	03 (7,5)	08 (20,0)	14 (35,0)	14 (35,0)
**15.** A entrega da minha parceria ao prazer sexual (“se soltar”).	02 (5,0)	02 (5,0)	10 (25,0)	13 (32,5)	13 (32,5)
**16.** A maneira como minha parceria cuida de minhas necessidades sexuais.	02 (5,0)	05 (12,5)	10 (25,0)	09 (22,5)	14 (35,0)
**17.** A criatividade sexual da minha parceria.	04 (10,0)	03 (7,5)	12 (30,0)	12 (30,0)	09 (22,5)
**18.** A disponibilidade sexual da minha parceria.	03 (7,5)	04 (10,0)	10 (25,0)	12 (30,0)	11 (27,5)
**19.** A variedade das minhas atividades sexuais.	03 (7,5)	07 (17,5)	08 (20,0)	14 (35,0)	08 (20,0)
**20.** A frequência da minha atividade sexual.	05 (12,5)	05 (12,5)	12 (30,0)	06 (15,0)	12 (30,0)

## DISCUSSÃO

Este estudo traduziu e adaptou a *New Sexual Satisfaction Scale* para pessoas com HIV, criando uma ferramenta confiável que avalia a satisfação sexual nessa população, auxiliando profissionais de saúde a promover cuidados mais humanizados e eficazes.

Na validação de escalas, a participação de especialistas com formação e experiência é fundamental, especialmente em estudos com pessoas que vivem com o HIV. A avaliação rigorosa do conteúdo quanto à relevância e adequação garante a qualidade do instrumento. Pesquisas como as de Kim et al. ^(29)^ e Yafi et al. ^(30)^ reforçam a importância da análise crítica e da padronização para o uso clínico futuro da escala.

Na tradução de escalas previamente construídas, podem surgir discrepâncias culturais em termos específicos, tornando essencial a participação de especialistas na língua-alvo e de tradutores na análise. Durante a tradução da NSSS para o português brasileiro, ajustes terminológicos foram necessários, assim como ocorreu na adaptação para o chinês ^(16)^, para garantir a compreensão pela população-alvo.

A tradução e retrotradução por tradutores independentes são etapas essenciais na validação de escalas. A NSSS passou por esse processo para o português brasileiro, assim como o Índice de Função Sexual Feminina para pacientes com Câncer de Mama (FSFI-BC), validada para mulheres com câncer de mama, que apresentou excelente confiabilidade de conteúdo e interna ^(31)^.

O perfil dos participantes desta pesquisa, especialmente quanto à escolaridade, é semelhante ao encontrado em estudos sobre estigma e HIV no Brasil. A escolaridade demonstrou ser um fator importante para a compreensão dos itens da escala. Em ambas as etapas, observou-se uma significativa proporção de participantes com ensino médio incompleto, o que reforça a importância de adaptar os instrumentos à realidade educacional da população-alvo ^(32)^.

Na análise semântica, 51,2% dos participantes se declararam homossexuais e, no teste, 57,5%. Pessoas vivendo com HIV que são homo ou bissexuais enfrentam duplo estigma, como observado no Líbano, onde 73,4% apresentaram ao menos um transtorno mental ^(33)^. A inclusão da diversidade sexual e de gênero na validação de escalas é fundamental para garantir instrumentos inclusivos e eficazes ^(34)^.

### Limitações do estudo

O estudo utilizou metodologias apropriadas, porém apresentou limitações, como a amostragem por conveniência realizada em um único serviço, o que impossibilitou a análise de invariância entre grupos. A validação em diferentes contextos culturais exige a aplicação da escala em diversos cenários, com amostras maiores e mais representativas. Além disso, por ter sido conduzido exclusivamente com essa população, a escala necessita de validações adicionais para outros públicos. Embora o reconhecimento da necessidade de futuras validações seja relevante, o estudo restringiu-se à análise da validade semântica e de conteúdo, não abrangendo etapas fundamentais do processo psicométrico, como validade de constructo, validade fatorial, validade relacionada a outras variáveis, consistência interna e testes empíricos. Também é importante considerar o perfil da amostra de especialistas envolvidos e as limitações quanto à generalização dos resultados, mesmo entre pessoas vivendo com HIV.

### Contribuições para área da enfermagem, saúde ou política pública

O instrumento oferece uma ferramenta de avaliação sobre a satisfação sexual de pessoas que vivem com o HIV o que contribui para a abordagem da sexualidade por esses profissionais de forma mais integral e humanizada.

## CONCLUSÕES

A *New Scale Satisfaction Sexual* demonstrou validade de conteúdo, assegurada pela avaliação de especialistas, e validade semântica, verificada por meio do pré-teste com a população-alvo. Recomenda-se que estudos futuros investiguem sua aplicabilidade clínica em distintos contextos culturais, a fim de consolidar seu uso fundamentado em evidências.

## Data Availability

Não se aplica.
